# Helical Irradiation of the Total Skin with Dose Painting to Replace Total Skin Electron Beam Therapy for Therapy-Refractory Cutaneous CD4+ T-Cell Lymphoma

**DOI:** 10.1155/2013/717589

**Published:** 2013-09-23

**Authors:** Chen-Hsi Hsieh, Pei-Wei Shueng, Shih-Chiang Lin, Hui-Ju Tien, An-Cheng Shiau, Yueh-Hung Chou, Meng-Hao Wu, Jen-Yu Wang, Chi-Kuan Chen, Yu-Jen Chen

**Affiliations:** ^1^Division of Radiation Oncology, Department of Radiology, Far Eastern Memorial Hospital, No. 21, Section 2, Nanya S. Road, Banciao District, New Taipei City 220, Taiwan; ^2^Department of Medicine, School of Medicine, National Yang-Ming University, Taipei 112, Taiwan; ^3^Institute of Traditional Medicine, School of Medicine, National Yang-Ming University, Taipei 112, Taiwan; ^4^Department of Radiation Oncology, Tri-Service General Hospital, National Defense Medical Center, Taipei 114, Taiwan; ^5^Department of Hematology, Far Eastern Memorial Hospital, New Taipei City 220, Taiwan; ^6^Department of Anatomical Pathology, Far Eastern Memorial Hospital, New Taipei City 220, Taiwan; ^7^Department of Radiation Oncology, Mackay Memorial Hospital, Taipei 104, Taiwan; ^8^Department of Dermatology, Mackay Memorial Hospital, Taipei 104, Taiwan; ^9^Department of Surgical Pathology, Mackay Memorial Hospital, Taipei 104, Taiwan

## Abstract

A 36-year-old woman was diagnosed with a therapy-refractory cutaneous CD4+ T-cell lymphoma, T3N0M0B0, and stage IIB. Helical irradiation of the total skin (HITS) and dose painting techniques, with 30 Gy in 40 fractions interrupted at 20 fractions with one week resting, 4 times per week were prescribed. The diving suit was dressed whole body to increase the superficial dose and using central core complete block (CCCB) technique for reducing the internal organ dose. The mean doses of critical organs of head, chest, and abdomen were 2.1 to 29.9 Gy, 2.9 to 8.1 Gy, and 3.6 to 15.7 Gy, respectively. The mean dose of lesions was 84.0 cGy. The dosage of left side pretreated area was decreased 57%. The tumor regressed progressively without further noduloplaques. During the HITS procedure, most toxicity was grade I except leukocytopenia with grade 3. No epitheliolysis, phlyctenules, tumor lysis syndrome, fever, vomiting, dyspnea, edema of the extremities, or diarrhea occurred during the treatment. HITS with dose painting techniques provides precise dosage delivery with impressive results, sparing critical organs, and offering limited transient and chronic sequelae for previously locally irradiated, therapy-refractory cutaneous T-cell lymphoma.

## 1. Introduction

Total skin electron beam therapy (TSEBT) is an effective treatment for cutaneous T-cell lymphoma affecting the superficial region [1]. One of the widely used techniques TSEBT is Stanford 6-dual field technique [2]. However, the dose in homogeneity is reported by the literatures [3, 4]. To improve this condition, a selection of patients with advanced skin disease and regional extension could be overcome by a combination of TSEBT and photon beam irradiation [5].

Helical tomotherapy (HT) has advantages in irradiating extended fields with dose painting techniques. Total marrow irradiation (TMI) via HT with low toxicities for multiple myeloma patients could be feasible [6]. According to the characteristics of HT, it is workable and feasible to replace conventional TSEBT technique by HT to increase dose homogeneity and decrease toxicities. Here, we report a successful case of therapy-refractory cutaneous CD4+ T-cell lymphoma treated with helical irradiation of the total skin (HITS) and dose painting technique to overcome the surface dose in homogeneity of conventional radiotherapy and to spare the previous irradiating area. Additionally, the data of surface dose, critical organs doses, and registration were analyzed too. 

## 2. Materials and Methods

### 2.1. Patient Characteristics

**Figure 1:**
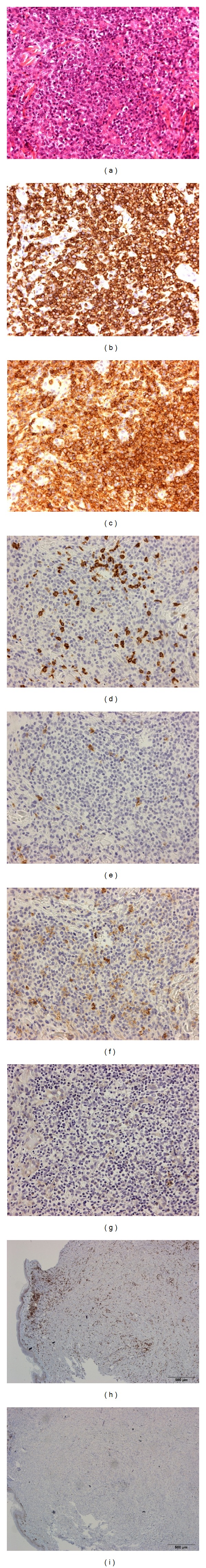
Pathology reports. (a) Atypical lymphoid cells infiltrated diffusely into the superficial and deep dermis. (b) Most of the atypical lymphoid cells were positive for CD3 (×200). (c) Most of the atypical lymphoid cells were positive for CD4 (×200). (d) Only a small portion of them were positive for CD8 (×200). (e) Only a small portion of them were positive for CD79a (×200). (f) Only a small portion of them were positive for CD56 (×200). (g) All negative for CD30 (×200). (h) Negative for CD3 (×40). (i) Negative for CD20 (×40) showed inflammation change without residual T-cell lymphoma.

In February, 2012, a 36-year-old woman visited our outpatient department due to the progression of a skin disease for several months. Eight months before visiting, she found a pruritic, noduloplaque skin rash over her trunk and extremities. Concurrently, a growing, fungating lesion 15 cm in diameter was in the left lateral chest wall. She visited one of medical center in Taiwan for help. The Ga-67 study showed intense uptake in the lateral left chest wall that was corroborated by the clinical appearance. A whole abdominal computer tomography (CT) showed several subcentimeter lymph nodes in the bilateral inguinal areas, and a biopsy was done. The pathology reports showed cutaneous CD4+ T-cell lymphoma, T3N0M0B0, and stage IIB without lymph node metastasis. Many medium- to large-sized atypical lymphoid cells infiltrated diffusely into the superficial and deep dermis (Figure 1(a)). Most of the atypical lymphoid cells were positive for CD3 (Figure 1(b)) and CD4 (Figure 1(c)). Only a small portion of them were positive for CD8 (Figure 1(d)), CD79a (Figure 1(e)), and CD56 (Figure 1(f)). They were all negative for CD30 (Figure 1(g)).

The prescriptions were interferon alpha, psoralen plus ultraviolet A photochemotherapy, and Accutane (Isotretinoin). In addition, local electron radiotherapy was delivered to the left chest wall and right axillary area with 50 Gray (Gy) in 25 fractions, respectively. After local radiotherapy, the producing newly-formed plaques over the trunk and buttock outside the radiation field were noted. Oral methotrexate (2.5 mg) 5 mg twice per day was prescribed immediately but disease progressed. The patient was referred to our hospital for total skin irradiation.

### 2.2. Regiment of Helical Irradiation of the Total Skin (HITS)

**Figure 2:**
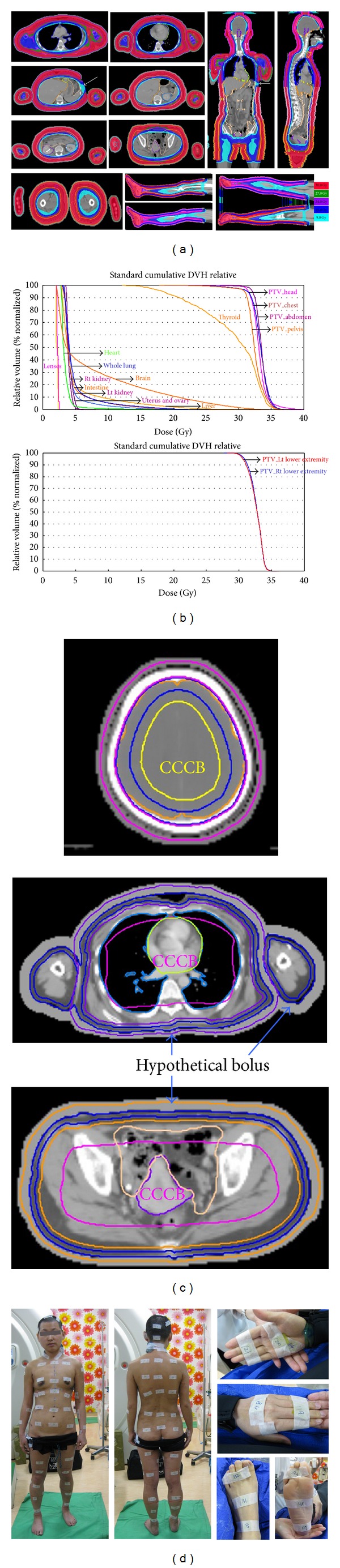
Irradiation techniques. (a) Helical irradiation of the total skin (HITS) with dose painting technique for total skin irradiation (white arrow indicates dose painting on the previous irradiation area). (b) The dose volume histograms (DVHs) of the target and individual organs at risk (OARs). (c) The hypothetical bolus was placed on the skin surface with 1–1.5 cm. A central core complete block (CCCB) 2.5 cm away from PTV in HITS planning. (d) The locations of radiochromic EBT2 film on the body surface.

HITS with dose painting techniques were applied from head to toe and avoided the previously treated areas. (Figures 2(a) and 2(b)) The patient was dressed with the diving suit (3 mm thick) to increase the superficial dose. The Polyflex II tissue equivalent material (Sammons Preston, Warrenville, IL, USA) was used as bolus for lesions over ears, fingers, and toes. The conformal bolus (R.P.D., Albertville, MN, USA) was used to cover the lesions in trunk. BlueBag immobilization system (Medical Intelligence, Germany) and thermoplastic fixation were used to fix head and neck, main trunk, and extremities. For tomotherapy treatment planning, a computed tomography (CT) image set of the whole body was required. The patients were scanned in a large bore (75 cm) CT scanner (GE, Discovery VCT PET/CT Imaging System) from head to toe. The level at 15 cm above knee was used as a reference point to separate the upper and lower set. The geometric edges of both fields were abutted at the HT treatment's 50% isodose plane. 

Both image sets were using the Philips Pinnacle^3^ treatment planning system for contouring. After that, the plan was transferred to the Tomotherapy *Hi Art *Planning system (v. 4.0.4. Tomotherapy, Inc., Madison, Wisconsin, USA). The clinical target volume (CTV) included the entire body surface system with subcutaneous 0.5 cm. To account for setup variability and respiratory motion, a planning target volume (PTV) was generated with a 0.5 cm margin at first. After 4 days treatment, the data of MVCT showed 0.5 cm for PTV was insufficient for some parts of body, such as shoulder, chest, abdomen, and pelvis. Therefore, the margins for PTV in these areas were changed accordingly. The anterior margin of the chest and abdomen was 1.0 cm with two-dimensional expansion and the shoulder was 0.8 cm with three-dimensional expansion, respectively. The CTV and PTV were sepacrated into five parts of head, chest, abdomen, pelvis, and upper extremities for the body plan. The hypothetical bolus was 1.0–1.5 cm in thickness from skin surface. Five mm was setting on the outer layer of PTV as hypothetical boluses during HITS plan to avoid the overhit of the inverse planning. Due to the different PTV margin used in the different part of body, the thicknesses of hypothetical boluses were variable. A central core complete block (CCCB) 2.5 cm away from PTV in HT planning system was used to restrict the photon beams to be an oblique incidence for increasing the superficial dose and reducing the internal organ dose (Figure 2(c)). 

Thirty Gy with 40 fractions interrupted at 20 fractions with one week resting, 4 times per week were prescribed. Total doses of 30 Gy to 95% of the PTV were delivered to the total skin area and tumor part, respectively. The normal tissue dose constraints utilized were based on the results of the survey of the clinical outcome of the target dose and dose limits to various organs at risk (OARs). The field width, pitch, and modulation factor (MF) used for the treatment planning optimization were 2.5, 0.287 cm, and 3.5, respectively. The dose volume histograms (DVHs) were calculated for the target and individual OARs. Toxicity of treatment was scored according to the Common Terminology Criteria for Adverse Events v4.0 (CTCAE v4.0).

### 2.3. Image Guidance

Daily check of patient positioning was performed by the megavoltage CT (MVCT) system integrated in the tomotherapy machine. MVCT scan from head to thigh were performed to check the patient's whole body alignment. Image fusions were evaluated by the attending physician and physicist. Any translational shifts suggested by the image fusion results were applied to the final patient setup before treatment delivery. The tolerance of setup error allowed only a 5 mm difference in any of the three translation directions and 1° of difference in roll. Additional selected MVCT scans were performed after treatment to verify patient immobilization.

### 2.4. Dose Measurement

**Figure 3:**
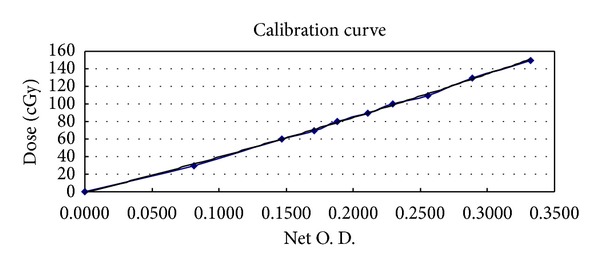
Calibration curve of radiochromic EBT2 film.

Radiochromic EBT2 film (International Specialty Products Inc. Wayne, NJ, USA) with thickness of 0.234 mm and effective measurement depth of 0.153 mm in a layer was used for dose measurements during HITS. Each film sheet was cut into smaller pieces as 5 × 5 cm that were placed on the lesions, head, chest, abdomen, pelvis, back, and extremities for calibration and measurement. The starting day in first period, 128 EBT2 films were measured (Figure 2(d)). In the starting day of second period, only 69 EBT2 films were putting on the important area of body to confirm the previous data. An Epson Perfection V700 flatbed scanner (Epson Seiko Corporation, Epson Seiko Corporation, Nagano, Japan) with the software of ImageJ Version 1.43 (National Institute of Health, Bethesda, MD, http://rsb.info.nih.gov/ij/) was used to scan all of the films at least 24 hours after film exposure. Films were scanned at a central scanner location and with the same orientation. The settings used were 48 bit color and 150 dpi (0.017 cm per pixel). Calibration was performed by irradiating each calibration film individually in a plastic water phantom perpendicularly to a 6 MV beam at dose levels from 0 to 150 cGy. The calibration curve was fitted using a polynomial function with the pixel value (PV) for each measurement film converted to dose accordingly (Figure 3). According to the calibration curve, the dose of the exposued EBT2 films can be measured.

## 3. Results

### 3.1. Response and Toxicities

**Figure 4:**
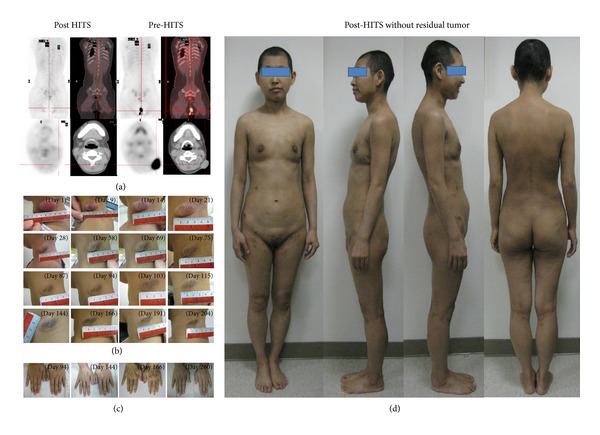
Treatment results. (a) Positron emission tomography study for tumor. (b) The tumor regressed progressively over the entire body without further noduloplaques. (c) Onycholysis during the two months following completion of the treatment. (d) The whole view of total body, transient alopecia was noted during HITS but she recovered without permanent partial alopecia 3 months later.

**Figure 5:**
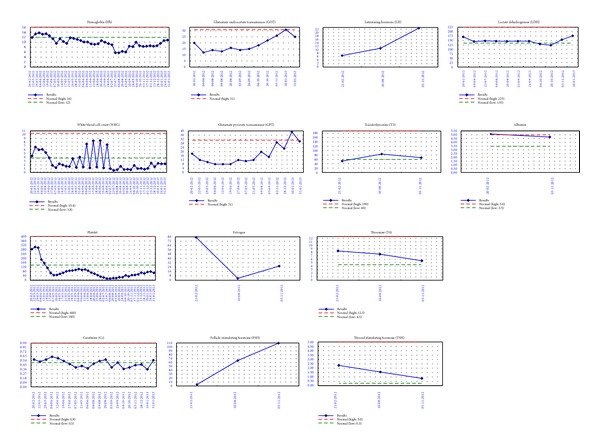
Laboratory results. No abnormal liver, renal, thyroid functions, or gonadotropin hormone were noted. Grade I anemia, thrombocytopenia, and grade 3 leukocytopenia were noted during the HITS procedure. Two months later, she developed grade 4 pancytopenia but recovered to grade 3 leukocytopenia and thrombocytopenia in the 3rd month after the treatment was completed.

Patient data was collected with the approval of the Institutional Review Board of our Hospital. Thirty gray were delivered to the patient from March 19, 2012 to June 29, 2012. The tumor regressed progressively over the entire body without further noduloplaques (Figures 4(a), 4(b), and 4(d)). After HITS, the following pathologic report showed only inflammation change without tumors persist (Figures 1(h) and 1(i)). Grade I dermatitis, mucositis, xerostomia, fatigue, and body weight loss (51 to 46 kg) were noted during the HITS and onycholysis during the two months following completion of the treatment (Figure 4(c)). Additionally, grade I anemia, thrombocytopenia, and grade 3 leukocytopenia were also noted during the HITS procedure (Figure 5). No epitheliolysis, phlyctenules, tumor lysis syndrome, fever, vomiting, dyspnea, edema of the extremities, or diarrhea occurred during the treatment. No abnormal liver, renal, thyroid functions, or gonadotropin hormone were noted during or after treatment (Figure 5). Transient alopecia was noted during HITS but she recovered without permanent partial alopecia 3 months later. Skin itching off and on over the trunk persisted from the beginning until the final report. Two months later, she developed grade 4 pancytopenia but recovered to grade 3 leukocytopenia and thrombocytopenia in the 3rd months after the treatment was completed. Supportive measures were provided including hematopoietic colony-stimulating factors (CSF), steroids, antioxidants, oral glutamine, and yeast-derived 1,3/1,6 glucopolysaccharide. From the treatment until now, a complete response was noted, with the white cell count recovering to grade 2, and the hemoglobulin and platelet counts recovering to grade 1 (Figure 5). 

### 3.2. Dosage of Organs at Risk (OARs)

Isodose distributions and dose volume histogram to the target and OARs were shown in Figures 2(b) and 2(c). The mean doses of HITS to various OARs of head, chest, and abdomen were 2.1 to 29.9 Gy, 2.9 to 8.1 Gy, and 3.6 to 15.7 Gy, respectively (Table 1(a)). 

### 3.3. Surface Doses and the Data of Registration

The surface doses in skin were listed in Table 1(b). The mean dosage of lesions was 84.0 cGy (ranged 73.6 to 89.4 cGy). The average dosage of left side pretreated area was 32.5 cGy. In here, the dose was decreased 57%. The average beam-on time for the upper and lower part took roughly 48.1 ± 7.9 min and 8.1 ± 0.8 min, respectively. The maximum average value of registration for upper torso versus lower extremities in different translation directions were 2.8 mm versus 0.9 mm for pretreatment and 0.7 mm versus 0.6 mm for posttreatment, respectively (Table 1(c)).

## 4. Discussion

This is a case of cutaneous T-cell lymphoma patient refractory to multiple modality therapies with disease progression then search for further management to avoid previous irradiation area. TSEBT could be an efficient and tolerable palliative treatment for patients with cutaneous manifestations of advanced, therapy-refractory cutaneous T-cell lymphoma [7]. Consensus guidelines for delivery of TSEBT have been published by the European Organization for Research and Treatment of Cancer (EORTC) [8]. The EORTC recommends a total dose of 31 to 36 Gy prescribed to the skin surface to produce a dose of at least 26 Gy at a depth of 4mm in the truncal skin along the central axis [8]. In the report by Anacak and colleagues, data of thermoluminescent dosimetry (TLD) measurements for TSEBT demonstrated that the dose in homogeneity throughout the skin surface is around 15% [9]. However, for less radiosensitive skin lymphomas, ±15% in homogeneity is not acceptable and innovative techniques are required. In the current study, the doses were 110% (82.5 cGy) at the surface and 100% (75 cGy) at a depth of 1 cm in the HITS plan (Figures 2(a) and 2(b)), sparing the previously treated area and fitting the requirement of recommendtions with high homogeneity.

The deviations of up to 40% occur from the prescription dosage and the surface dose in homogeneity can vary as much as 90% in body areas such as the perineum and eyelid [3]. Additionally, cutaneous tumors often exceed the 4 mm depth and are consequently underdosed when treated with TSEBT alone. HT has advantages in irradiating extending area that make it possible to replace total body irradiation with total marrow irradiation, lowering the toxicities, and sparing critical organs [6]. Using these characteristics of HT, HITS provides dose homogeneity with precise depth penetration and decreased toxicities. In the current study, the CTV included the entire body surface system with subcutaneous 0.5 cm. The maximum average value of registration for upper torso versus lower extremities in different translation directions were 2.8 mm versus 0.9 mm for pretreatment and 0.7 mm versus 0.6 mm for posttreatment, respectively. Generous PTV with a 0.5 cm margin, the shoulder with 0.8 cm margin, and the anterior of the chest and abdomen with 1.0 cm margin was acceptable for compensating the clinical setup variability and breathing motion (Table 1(c)). The hypothetical bolus was designed during HITS plan to avoid overhitting. Diving suit and actual boluses were used in daily practice to increase surface dose. Both hypothetical and actual boluses contributed to skin doses of whole body delivering no more than 125% and no less than 95%. The lowest and highest doses of measurement were located in left anterior lower leg (73.2 cGy) and upper-middle chest (92.6 cGy), respectively. The mean dosage of lesions was 84.0 cGy and the dosage of left side pretreated area was decreased 57% that was matched with the planning expectation (Table 1(b)). Now we have the possibility of replacing conventional TSEBT with HITS with dose painting technique, while still achieving encouraging results (Figures 1(h), 1(i), 4(a), 4(b), and 4(d)). 

Common acute toxicities from TSEBT include pruritus, dry desquamation, erythema, alopecia, xerosis, bullae of the feet, edema of the hands and feet, hypohidrosis [10], hyperpigmentation of the skin [7], phlyctenules [5], and loss of fingernails and toenails [11, 12]. Rare acute side effects include gynecomastia in men and mild epistaxis or parotitis [11]. Long-term complications are typically mild and may include permanent nail dystrophy, xerosis, telangiectasias, partial scalp alopecia, and fingertip dysesthesias [8]. Young patients should be thoroughly counseled regarding risks of gonadal toxicity [13]. In addition, a grade 3 erythema with bullous reaction during TSEBT was recorded in 26–32% of the cases [5, 7, 14]. The central CCCB techniques in HITS planning restrict the photon beams delivering obliquely and reduced doses of OARs (Figure 2(c)). The mean doses of thyroid, lung, liver, right kidney, left kidney, intestine, and uterine and ovary were 24.7, 4.6, 5.2, 3.9, 4.3, 4.3, 4.7, and 4.3 Gy, respectively (Table 1(a)). On the other hand, most of the toxicities during and after HITS are grade 1 with complete recovery. No destruction of the liver, renal, thyroid or gonadotropin functions was noted (Figure 5). 

For TSEBT combined with photon beam irradiation, hematological complications related to the photon were a concern. Maingon and colleagues [5] noted myelosuppression (grade 2 WHO) in 17 cases. Additionally, after total body irradiation, 2/20 patients had a neutropenia below 500 granulocytes reversible without complication. Using HITS techniques, the hematological complications were reversible. Hematopoietic damages were treated with supportive measures including hematopoietic CSF [15], steroids [16], antioxidants [17], oral glutamine [18], and yeast-derived 1,3/1,6 glucopolysaccharide [19] to stimulate the granulocytes (neutrophils and eosinophils), monocytes, macrophages, and NK cell production and to modulate the immune system. The mean doses to the bone marrows (BMs) of the cervical, thoracic, and lumbar spine, sacrum, and bilateral iliac bone were 5.8, 6.3, 4.0, 4.8, and 8.7 Gy, respectively. In the future, the constraints for these BMs in the HITS plan should be stricter to diminish the possibility of hematological damages. 

## 5. Conclusion

To our best knowledge, this is the first report of cutaneous T-cell lymphoma treated with HITS techniques to alternate TSEBT. HITS with dose painting techniques provide precise dosage delivery with impressive results, sparing critical organs, and offering limited transient and chronic sequela for advanced, previously locally irradiated, therapy-refractory cutaneous T-cell lymphoma. The proposed technique could be considered an acceptable alternative to TSEBT once the techniques are further improved to avoid the hematologic complications. Long-term followup is needed to confirm these preliminary findings.

## Figures and Tables

**Table 1a:** (a)

Critical organ	D10 (Gy)	D90 (Gy)	Mean dose (Gy)
Whole brain	21.5	2.3	8.0
Brain stem	3.0	2.2	2.5
Spinal cord	4.1	3.5	3.8
Right lens	2.2	2.1	2.1
Left lens	2.3	2.1	2.2
Right eye	7.7	2.1	3.8
Left eye	6.9	2.3	4.1
Right parotid gland	33.8	17.5	29.3
Left parotid gland	33.7	21.7	29.9
Lips	21.8	11.0	15.8
Oral cavity	19.8	2.6	8.7
Pharynx	11.1	2.5	5.2
Larynx	31.8	11.8	23.2
Trachea	29.9	3.1	13.7
Thyroid	31.5	17.2	24.7
Esophagus-upper part	15.2	3.3	8.1
Esophagus-middle part	3.1	2.9	3.1
Esophagus-lower part	2.9	2.8	2.9
Right lung	6.9	3.2	4.7
Left lung	5.9	3.2	4.5
Whole lung	6.4	3.2	4.6
Heart	3.9	2.8	3.3
Liver	9.7	2.9	5.2
Spleen	15.0	3.6	6.8
Right kidney	4.4	3.3	3.9
Left kidney	5.1	3.3	4.3
Bladder	16.9	5.5	11.2
Rectum	20.4	4.1	8.5
Uterus and ovary	5.4	3.7	4.3
Cervix and vagina	25.5	4.4	15.7
Intestine	6.8	3.3	4.7
Stomach	4.3	3.0	3.6
Cervical spine	10.8	3.2	5.8
Thoracic spine	17.6	3.2	6.3
Lumbar spine	4.5	3.2	4.0
Sacrum	7.8	3.4	4.8
Right iliac crest	29.8	3.6	8.9
Left iliac crest	26.9	3.5	8.5
Right femur	31.4	6.9	12.3
Left femur	14.1	6.7	10.3
Right pelvic bone	23.1	3.7	13.1
Left pelvic bone	22.5	3.5	12.2

D10: The dose received to 10% of the organ volume.

D90: The dose received to 90% of the organ volume.

**Table 1b:** (b)

Site	Surface dose (cGy)/fraction			Percentage of prescription dose
	1st measurement	2nd measurement	Average	
Head				
Vertex of head	77.2	74.7	76.0	101.3%
Occipital	82.4	80.6	81.5	108.7%
Forehead	76.2	83.7	80.0	106.6%
Neck				
Anterior	73.2	73.9	73.6	98.1%
Posterior	99.8	85	92.4	123.2%
Chest				
Middle, upper	94.6	90.5	92.6	123.4%
Middle, lower	94.7	86.3	90.5	120.7%
Right axillary	89.1	89	89.1	118.7%
Left axillary	88.9	89.8	89.4	119.1%
Previous treated area (Left flank)	32.1	32.9	32.5	43.3%
Back				
Middle, upper	88.4	85.4	86.9	115.9%
Middle, lower	92.2	85.4	88.8	118.4%
Abdomen				
Middle, anterior	88.9	90.4	89.7	119.5%
Middle, posterior	99.4	86.3	92.9	123.8%
Upper extremities, right				
Upper arm	76.7	81.8	79.3	105.7%
Elbow	97.3	86.1	91.7	122.3%
Hand	84.8	80.1	82.5	109.9%
Fingers	84.7	87.1	85.9	114.5%
Upper extremities, left				
Upper arm	81.1	87.9	84.5	112.7%
Elbow	88.2	90.8	89.5	119.3%
Hand	86.2	84.5	85.4	113.8%
Fingers	85.3	87	86.2	114.9%
Right side of lower extremities				
Thigh, anterior	88	76.8	82.4	109.9%
Thigh, posterior	87.5	70.9	79.2	105.6%
Thigh, medial	88.8	76.1	82.5	109.9%
Lower leg, anterior	80.8	70.4	75.6	100.8%
Lower leg, posterior	82.3	64	73.2	97.5%
Foot	78	77.8	77.9	103.9%
Toes	90.5	88.9	89.7	119.6%
Left side of lower extremity				
Thigh, anterior	86.1	74.3	80.2	106.9%
Thigh, posterior	99.6	82.4	91.0	121.3%
Thigh, medial	78	84.7	81.4	108.5%
Lower leg, anterior	81.9	61.7	71.8	95.7%
Lower leg, posterior	86.7	67.1	76.9	102.5%
Foot	79.1	83.2	81.2	108.2%
Toes	89.3	90.2	89.8	119.7%
Lesions				
Right ear lesion	85.5	74.6	80.1	106.7%
Left ear lesion	83.5	89	86.3	115.0%
Right neck lesion	92.1	78.3	85.2	113.6%
Lift neck lesion	94.4	76.7	85.6	114.1%
Buttock	75.8	71.3	73.6	98.1%
Vulva	89.5	82.4	86.0	114.6%
Right abdominal mass	91.5	87.2	89.4	119.1%
Right wrist	91.8	80.0	85.9	114.5%

**Table 1c:** (c)

Shift	Pretreatment		Posttreatment	
	Upper torso	Lower extremities	Upper torso	Lower extremities
Lateral (mm)	−2.76 ± 0.75	0.86 ± 1.18	−0.24 ± 0.37	0.61 ± 1.41
Longitudinal (mm)	0.72 ± 2.29	−0.01 ± 2.04	−0.74 ± 1.61	0.22 ± 2.18
Vertical (mm)	0.18 ± 2.17	−0.06 ± 1.99	0.28 ± 0.67	0.09 ± 1.00
Roll (degree)	0.14 ± 0.28	0.09 ± 0.10	−0.16 ± 0.32	−0.03 ± 0.09
